# Initial experience with isotropic 3D cardiac T_2_ mapping for the monitoring of cardiac allograft rejection

**DOI:** 10.1186/1532-429X-18-S1-W23

**Published:** 2016-01-27

**Authors:** Ruud B van Heeswijk, Hélène Feliciano, Mélanie Metrich, Davide Piccini, Samuel Rotman, Juerg Schwitter, Roger Hullin

**Affiliations:** 1 CardioVascular MR Research Group (CVMR), Department of Radiology, University Hospital (CHUV) and University of Lausanne (UNIL), Lausanne, Switzerland, Lausanne, Switzerland; 2 Cardiology Service, Department of Internal Medicine, University Hospital (CHUV) and University of Lausanne (UNIL), Lausanne, Switzerland; 3 Advanced Clinical Imaging Technology, Siemens Healthcare IM BM PI, Lausanne, Switzerland; 4 Institute of Pathology, University Hospital (CHUV) and University of Lausanne (UNIL), Lausanne, Switzerland; 5 grid.8515.90000000104234662Center for Cardiac Magnetic Resonance (CRMC), University Hospital of Lausanne (CHUV), Lausanne, Switzerland

## Background

Cardiac T_2_ mapping has been suggested for monitoring of acute allograft rejection, since the T_2_ relaxation time increases with myocardial edema [1]. Besides its non-invasive nature, the main advantage of T_2_ mapping over the reference standard endomyocardial biopsy (EMB) is that it results in a higher spatial coverage of the myocardium. Currently established 2D techniques are used to acquire several slices in short- and long-axis orientation, which should suffice for the detection of moderate to severe rejection (ISHLT degree 2R-3R [2]), since the manifestation of edema is global. However, in the case of the more common mild rejection, the manifestation of edema is localized and patchy, and might thus be missed by a selective 2D visualization. We therefore investigated the performance of a novel 3D cardiac T_2_ mapping technique [3] for the detection of acute allograft rejection versus 2D T_2_ mapping and EMB.

## Methods

28 Patients (age 54 ± 12 y, 24 males) underwent routine EMB as well as 2D and 3D cardiac T_2_ mapping at 3T. Navigator-gated 2D T_2_ maps [4] (voxel size 1.2 × 1.2 × 5 mm^3^) in 3 short-axis slices and a prototype self-navigated 3D radial whole-heart isotropic T_2_ map [3] (voxel size 1.7 mm^3^) were acquired with 3 T_2_-preparation durations and free breathing. After reformatting of the 3D T_2_ maps and matching for slice thickness, the 2D and 3D T_2_ maps at the same location were segmented according to AHA guidelines [5]. The highest segmental 2D and 3D T_2_ values of each patient were compared statistically, and then divided into groups according to their EMB rejection degree. These groups were then tested for differences in T_2_ value. The 3D T_2_ maps were furthermore directly rendered in 3D, after which they were inspected for foci of T_2_ elevation.

## Results

**Figure 1:**
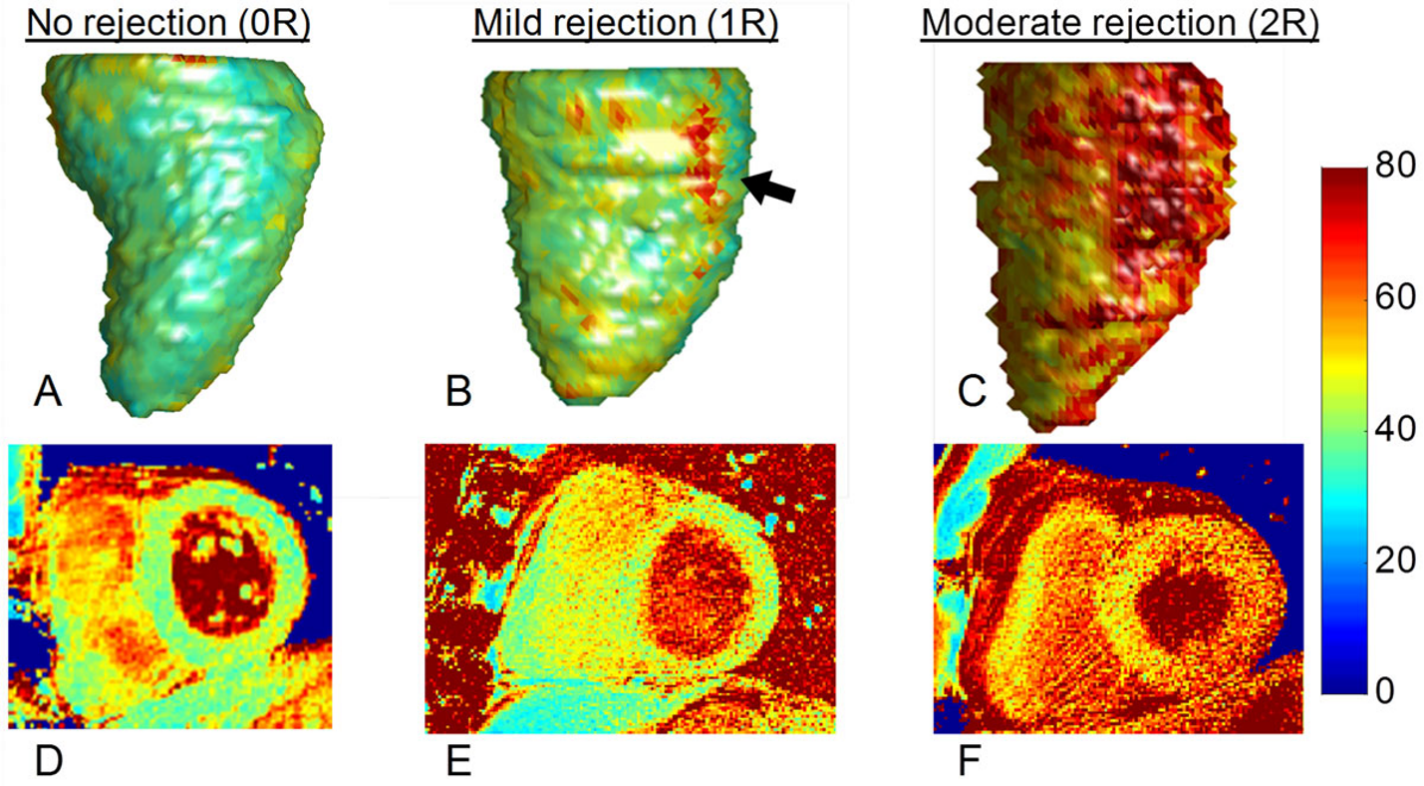
**3D & 2D T**_**2**_ **maps of cardiac allograft rejection**. **A-C)** Examples of rendered 3D T_2_ maps that were segmented along the center of the endocardium. **D-F)** Corresponding basal 2D T_2_ maps. While the segmental T_2_ values in 2D T_2_ maps of the patients with mild rejection as determined through EMB were not elevated, the corresponding 3D T_2_ maps contained myocardial regions with significantly elevated T_2_ values (black arrow). The color bar indicates T_2_ values in ms for all maps.

EMB analysis indicated allograft rejection in 3 out of 28 cases (i.e. 25 × 0R, 2 × 1R and 1 × 2R). The highest 2D segmental T_2_ values of the groups were 49.9 ± 4.0 ms (0R), 48.9 ± 0.8 ms (1R), and 65.0 ms (2R). The reformatted 3D T_2_ values agreed very well with the 2D T_2_ values for all patients (p = 0.84, Figure 1). While neither of the 1R cases demonstrated significantly elevated segmental T_2_ in the 2D or 3D T_2_ maps, foci of elevated T_2_=58.2 ± 3.6 ms that were not visible on the 2D T_2_ maps could be clearly identified in both their rendered 3D T_2_ maps (Figure 1B, black arrow).

## Conclusions

The investigated 3D cardiac T_2_ mapping agreed with the established 2D technique, and enables the identification of foci of elevated T_2_ in regions of the myocardium that are not covered by the 2D technique. The 3D cardiac T_2_ mapping technique thus appears to be well-suited for the investigation of mild allograft rejection (degree 1R), but this remains to be confirmed in a larger patient cohort.
